# Synbiotics in Children with Cow's Milk Allergy: A Randomized Controlled Trial

**Published:** 2014-01-03

**Authors:** Hamid Ahanchian, Zahra Nouri, Seyed-Ali Jafari, Toktam Moghiman, Mohammad-Hadi Amirian, Atefeh Ezzati, Hamid-Reza Kianifar

**Affiliations:** 1 Department of Pediatric Allergy and Immunology; 2 Research Development Center, Ghaem hospital; 3 Department of Pediatric Gastroenterology; 4 Department of Pediatrics, Mashhad University of Medical Sciences, Mashhad, Iran

**Keywords:** Infant, Cow`s Milk Protein Allergy, Symbiotic, Children

## Abstract

***Objective:*** Cow`s milk protein allergy usually occurs in infants within the first months of life. It can affect several organs, but gastrointestinal symptoms are the most clinical symptoms observed. The most effective treatment is restricting the cow `s milk protein in mother and infant`s diet. Lactobacillus GG supplementation in infant could be effective through modulation of the immune system and the gut microflora.

***Methods:*** Thirty two breastfed infants with cow`s milk protein allergy were enrolled in a double-blinded randomized controlled trial in which they received Synbiotic (n=16) or placebo (n=16) once a day for one month, simultaneously with cow`s milk protein restriction in mother and infant`s diet. Clinical gastrointestinal symptoms (vomiting, colic, rectal bleeding and diarrhea), head circumference, body length and weight were recorded at the beginning, the end of the first and third month of study.

***Findings*** ***:*** Percentage of increment in head circumference and weight were statistically more in synbiotic group compared with placebo group at the end of the first and third month of study. There was no significant difference in resolution of clinical gastrointestinal symptoms (vomiting, colic, rectal bleeding or diarrhea) and percentage of increment in body length.

***Conclusion:*** Synbiotic supplementation in infants may improve increment of head circumference and weight gain, but has no effect on resolution of clinical symptoms.

## Introduction

Allergic diseases, especially food allergy, have been on a rise in the recent years^[^^1^^]^. An estimated 10%-15% of the population report symptoms of food allergy^[^^2^^] ^whereas the actual prevalence of cow's milk allergy (CMA) in children varies and seems to range between 0.1 and 4.2% in different countries worldwide^[^^3^^-^^4^^]^. 

 Cow's milk protein allergy is one of the most common allergies which is of great importance in many aspects. In addition to its clinical symptoms and limited diagnostic approach, when omitted from the child's daily diet causes major nutritional concerns in the child and occasionally in the mother.

 Therefore, primary prevention of cow's milk allergy, reducing clinical symptoms, disease duration and nutritional deficits are of special concern^[^^5^^]^. Recently, food allergy preventive approaches in pregnancy have shifted from avoidance towards exposure, because in some countries, mainly Australia, the occurrence of food allergy in infants whose mothers avoided common food allergens, was higher than in those whose mothers had a free daily diet^[^^6^^]^. As a good example, in comparison to introduction at 4–6 months, introducing egg into the infant’s diet later has been associated with higher rates of egg allergy^[^^7^^]^.

 On the other hand, the importance of the infantile immune system maturation especially in the first few months of life and its effect on developing allergic diseases makes these early years the ideal time for prevention of such diseases^[^^3^^,^^4^^,^^8^^,^^9^^]^. 

 Previous studies have proven the efficacy of probiotics on clinical symptoms of cow's milk protein allergy (CMPA)^[^^10^^-^^11^^] ^as well as several other diseases^[^^12^^-^^16^^]^, but some recent studies have shown no benefits^[^^17^^]^. As both prebiotics and probiotics have immunomodulation effects, their mixture as synbiotic may be more effective in tolerance induction. In this study we evaluated the effect of a newly introduced symbiotic, a mixture of seven probiotic bacteria and fructo oligo sacharide (FOS), on the infants’ growth rate, clinical symptoms and disease course of cow's milk protein allergy in this age group.

## Subjects and Methods

**Fig.1:**
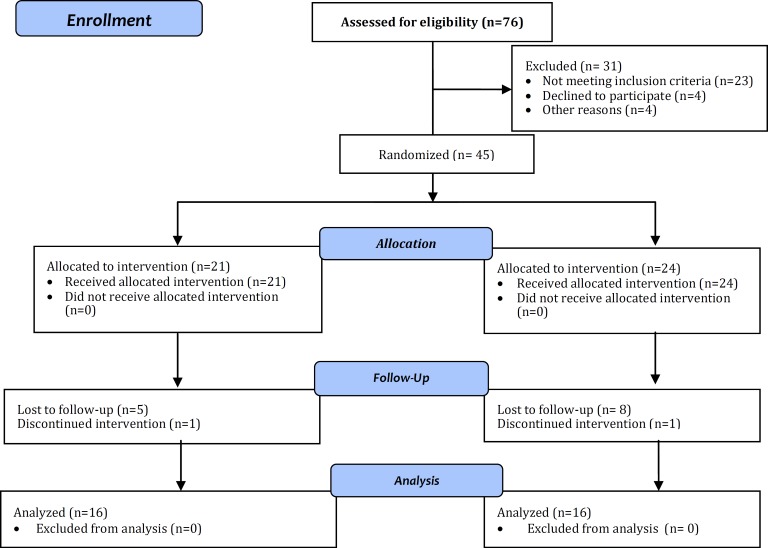
Flow diagram of the study

This was a randomized double blind clinical trial performed from February 2009 to December 2010 in the pediatric allergy and gastrointestinal clinics of Ghaem education and research center, Mashhad, Iran. Totally 32 infants were randomly divided into the study and placebo groups each consisting of 16 cases (Fig. 1). The inclusion criteria consisted of 1-12 month-old infants with the clinical symptoms of cow's milk protein allergy including rectal bleeding, diarrhea, vomiting and evidences of colitis with complete resolution of symptoms following exclusion of dairy products from diet of mother and infant and confirmed by reappearance of symptoms after reintroducing dairy products, a good general condition, breast milk feeding and cow's milk consuming mother.

 The infants were excluded from the study if they had a known immune deficiency, gastrointestinal disease, positive stool culture, coagulopathy or were receiving any type of antibiotic therapy in the past two weeks (Fig. 1).

**Table 1:** Baseline characteristics of the studied cases

**Baseline characteristics**	**Study group (SD)**	**Placebo group (SD)**	***P-*** **value**
**Age (month)**	6 (2.4)	5.5 (2.8)	0.3
**Sex**	5/11	8/8	0.2
**Head Circumference (cm)**	41.68 (2.25)	42.46 (1.91)	0.3
**Height (cm)**	66.93 (4.5)	65.9 (4.25)	0.5
**Weight (gr)**	7028 (1038.5)	7728 (1271.7)	0.1

SD: Standard Deviation

 The patients' data including name, clinical symptoms, growth indices (height, weight and head circumference) were recorded in a questionnaire on entering the study. At the end of the first 72 hours, first week, second week and third week the clinical symptoms were obtained by phone and recorded. At the end of the first and final month the patients visited at the child's GI clinic where their clinical symptoms and growth indices were recorded. Clinical diagnosis and follow up visits were performed by the same person.

 After fully explaining the study protocol and obtaining an informed consent from the child's guardian, a synbiotic containing 1 billion Colony Forming Units (CFU) of Protexin^R^ Restore: a mixture of *Lactobacillus casei, Lactobacillus rhamnosus, Streptococcus thermophilus, Bifidobacterium breve, Lactobacillus acidophilus, Bifidobacterium infantis, Lactobacillus bulgaricus* and FOS (Protexin healthcare, Somerset, UK), in the form of freeze dried powder was fed daily for 4 weeks.

 The placebo group received placebo with the same shape, color and packaging for the same duration. During this time the consumption of all kinds of food containing cow's milk protein was prohibited in all infants and their mothers. Ethics committee of Mashhad University of Medical Sciences has approved the proposal. 

 For data analysis the R software language was used. Shapiro-Wilk normality test, t-test, Wilcoxon rank sum test and Chi-square tests were applied and a *P-*value <0.05 was considered as statistically significant.

## Findings

Placebo and study groups, each included 16 cases, with mean age of 6 and 5.5 months, respectively. Baseline characteristics of the studied cases are shown in Table 1. At the beginning of study, all patients in both groups had diarrhea. Rectal bleeding was seen in 81.25% and 75% in placebo and study groups, respectively. 18.5% in placebo group and 25.5% in study group had colics as a symptom. 12.5% of patients in both groups had vomiting. There was no significant difference in reduction of the daily vomiting or diarrhea between case and placebo groups (Table 2 and 3). 

 During the study, there were no significant differences in rectal bleeding between two groups. Intestinal colic got better in both groups after 72 hours and no patient had colic after 2 weeks but the difference was not significant. After 1, 2, 3 and 6 moths follow up there was no significant difference between symptomatic patients (who had at least one symptom) in the two groups. 

Table 4 shows that at the end of the first and third month, the difference of head circumference weight between the two groups was statistically significant, but not significant for height.

**Table 2:** Comparing the mean reduction in daily vomiting between the study and placebo group

**Time **	**Study group**	**Placebo group**	***P. *** **value**
	**Mean (SD)**	**Mean (SD)**	
**After 72 hrs**	-0.25 (0.77)	-0.31 (0.84)	0.5
**End of week 1**	-0.25 (0.77)	-0.37 (1.09)	0.5
**End of week 2 to end of the 3** ^rd^ ** month**	-0.25 (0.77)	-0.75 (2.05)	0.6

SD: Standard Deviation

**Table 3:** Comparing the mean reduction in daily defecation times between the study and placebo group

** Time**	**Study group**	**Placebo group**	***P. *** **value**
	**Mean (SD)**	**Mean (SD)**	
**After 72 hrs**	-1.62 (1.15)	-1.87 (2.30)	0.4
**End of week 1**	-2.06 (1.29)	-2.56 (2.68)	0.4
**End of week 2**	-2.69 (1.25)	-2.97 (2.71)	0.3
**End of week 3**	-2.97 (1.60)	-3.56 (2.68)	0.8
**End of the 1** ^st ^ **month**	-3.38 (2.06)	-3.80 (2.76)	0.7
**End of the 2** ^nd ^ **month**	-3.83 (2.23)	-4.41 (2.92)	0.8
**End of the 3** ^rd ^ **month**	-4.15 (2.11)	-4.43 (2.77)	0.7

SD: Standard Deviation

## Discussion

In this clinical trial we showed that using synbiotics for four weeks in infants with symptoms of CMA may increase growth indices but had no significant effect on gastrointestinal symptoms and disease course.

 There are several studies about effect of probiotics on rapid resolution of allergy symptoms.

Vandenplas et al revealed that both casein and whey hydrosylates formula enriched with probiotics can improve symptom scores in cow’s milk allergy^[^^18^^]^. Baldassarre et al showed that adding *Lactobacillus GG* to extensive hydrolyzed formula compared to taking this type of formula alone in cow’s milk induced colitis can improve colonic symptoms as well as reducing stool calprotectin and occult blood^[^^19^^]^. Ivakhnenko et al in an open randomized prospective clinical trial using *Bifidobacterium lactis* *BB-12* (1х109 CFU) and *Streptococcus thermophilus* *TH-4* (1х108 CFU) for four weeks showed that probiotics in addition to elimination diet in children with atopic dermatitis and cow's milk allergy may decrease gastrointestinal symptoms including diarrhea, constipation and infantile colic^[^^20^^]^. According to our study, we could not find any significant reduction in diarrhea and colic symptoms between the two groups. Used strains in our study were different to those of Vandenplas and Baldassarrre’s. Doses and duration of probiotics were similar in our study and Ivakhnenko et al. Their sample size was larger than ours and it may explain the difference between these findings^[^^20^^]^. 

 The effect of probiotics on tolerance acquisition in patients with CMA is an unexplored area of research. Some studies have shown that supplementation of probiotics suppressed the allergic reaction mainly through increased intestinal secretary IgA and regulatory T cell induction^[^^21^^]^. West et al showed that a tolerogenic environment could be formed by dietary factors, including polyunsaturated fatty acids, probiotics, oligosaccharides, antioxidants, folic acid, and other vitamins such as vitamin D^[^^9^^]^. Once allergies are developed, the consumption of synbiotics may to some extent reduce symptoms and complications. In a recent interesting study, Canani et al showed supplementation of an extensively hydrolyzed casein formula with *Lactobacillus GG* accelerated tolerance acquisition to cow’s milk protein (CMP)^[^^11^^]^. 

**Table 4:** Changes of weight, height and HC in the studied cases after 1 and 3 months of follow up

** Variable **	**Study group** **Mean (SD)**	**Placebo group** **Mean (SD)**	***P. *** **value**
**Head circumference (after 1** ^st^ ** month)**	0.02 (0.01)	0.02 (0.01)	0.048
**Head circumference (after 3** ^rd^ ** month)**	0.06 ( 0.03)	0.05 (0.02)	0.03
**Height (after 1** ^st^ ** month)**	0.02 (0.01)	0.03 (0.02)	0.7
**Height (after 3** ^rd ^ **month)**	0.06 (0.03)	0.08 (0.05)	0.9
**Weight (after 1** ^st ^ **month)**	0.12 (0.07)	0.06 (0.06)	0.008
**Weight (after 3** ^rd ^ **month)**	0.25 (0.15)	0.15 (0.10)	0.02

SD: Standard Deviation

As our study design did not include long term follow up, we could not have a reliable judgment about tolerance acquisition in our patients. But increasing growth indices in our study may indicate improving disease state.

 Gut microbiota may play a role in weight gain by different mechanisms such as improvement of mineral bioavailability, synthesis of vitamins, regulation of gastrointestinal secretion and motility, digestion of macronutrients and regulation of energy extraction from the diet^[^^3^^,^^22^^,^^23^^]^. Some studies showed that changing microflora can increase weight gain^[^^24^^,^^25^^]^ but it was inconsistent in other studies^[^^26^^]^ taking into consideration several other factors including the probiotic type, dosage and the patient‘s age. In a recent meta-analysis, *Lactobacillus gasseri* was associated with weight loss both in obese humans and in animals, *Lactobacillus fermentum *and *Lactobacillus ingluviei* were associated with weight gain and *Lactobacillus plantarum* was associated with weight loss in animals. The authors concluded that different Lactobacillus species are associated with different effects on weight change that are host-specific^[^^27^^]^. This emphasizes that functional capabilities of probiotics are strain-dependent. In our study we could show that combinations of seven probiotics with fructooligosaccharides increase weight and other growth indices. 

 As the therapeutic effects of synbiotics depend on type of probiotic strains, single-strain or multi-strain, dosage, duration and study population, these variables are the main causes of different results in clinical trials^[^^12^^,^^28^^]^. Ideal commercial product should contain probiotics or synbiotic strains that along with tolerance induction can increase growth status as well. 

 Moreover, this study had its own limitations; the number of studied cases was not large enough.

 Future studies should be carried out on a greater number focusing on the early prescription of synbiotics (even during pregnancy), and at the critical age, first few months of life, when the tolerogenic environment can affect the incidence of allergic diseases. Finally, as different factors contribute to the development of food allergy and other types of allergies, a successful model of prevention should consider additional measures in the mother and child diet which affect tolerance induction including vitamin D status, antioxidants and folic acid^[^^9^^]^.

## Conclusion

This study showed that synbiotics may increase growth indices (weight and head circumference) in children with cow’s milk protein allergy.
